# Bile or Acid Reflux Post One-Anastomosis Gastric Bypass: What Must We Do? Still an Unsolved Enigma

**DOI:** 10.3390/jcm11123346

**Published:** 2022-06-10

**Authors:** Tigran Poghosyan, Sylvia Krivan, Clement Baratte

**Affiliations:** 1 Department of Digestive, Oncologic and Bariatric Surgery, Hôpital Européen Georges Pompidou, AP-HP, F-75015 Paris, France; clementbaratte@hotmail.fr; 2 Université de Paris Cité, F-75005 Paris, France; 3 2nd Department of General Surgery, Upper Digestive Tract Surgery Center, IASO Group (General Clinic), 15123 Athens, Greece; navirk@hotmail.com

Obesity is a global scourge, affecting over 15% of the world’s population [1]. Bariatric surgery combined with medical and dietetic input is the most effective treatment of morbid obesity. Among all certified surgical procedures, one-anastomosis gastric bypass (OAGB) is a promising therapeutic option [2]. Due to its technical straightforwardness, low early morbidity rates, as well as long-term efficacy on weight loss and comorbidity improvement, OAGB has become the third most commonly performed bariatric procedure worldwide over the last two decades [3]. This procedure involves a long and narrow gastric pouch, anastomosed to a jejunal loop, 200 cm from the angle of Treitz [4,5]. Despite the benefits observed, gastroesophageal reflux disease (GERD) and, to a lesser extent, malnutrition, are the two Achilles heels of this procedure and pose as the main reason for revisional surgery post OAGB [6,7,8]. The GERD rate after this procedure reported in the literature varies from 3 to 8% [6]. The main hypothesis to date is that of bile origin [9]. Its role in the genesis of gastritis, esophagitis, anastomotic ulcer, as well as gastric and esophageal cancer has been pointed out in several studies [10,11,12]. To begin with, it is essential to understand the mechanism of post-OAGB bile reflux occurrence, as there is confusion in the literature regarding its definition. It is mandatory to distinguish between reflux of bile into the gastric pouch and gastroesophageal reflux. It is common in clinical practice to come across gastroscopy reports that conclude in the presence of bile reflux into the gastric pouch post OAGB. The mechanism of bile reflux into the gastric pouch is easily conceived. Due to the surgical technique, the gastrojejunal anastomosis is permanently exposed to bile flow [13]. The absence of an anatomical barrier, such as a sphincter at the gastrojejunal junction, may allow the passage of bile into the gastric pouch and could promote the development of gastritis and marginal ulcer. This mechanism was highlighted in a recent study on bile reflux scintigraphy showing that transient bile reflux post OAGB is common in the gastric tube, but not in the esophagus [14]. In order to prevent bile reflux post OAGB, various solutions have been proposed such as the creation of a narrow (3–4 cm) and long (11–15 cm) gastric pouch, latero-lateral gastrojejunal anastomosis, and antireflux sutures with afferent loop suspension, 8–10 cm above the anastomosis [15]. However, just the presence of bile in the gastric pouch is not synonymous with GERD. The main predisposing factor in the development of GERD is the congenital or acquired insufficiency of the lower esophageal sphincter [12]. In case of lower esophageal sphincter insufficiency, bile backflows in the esophagus and promotes GERD symptoms. However, the hypothesis of bile reflux in all cases of GERD post OAGB cannot explain the effectiveness of proton pump inhibitors (PPI) in some patients. PPI administration is a standard treatment for acid reflux but in no way effective on non-acid reflux. For the first time, the presence of acid reflux in nearly one out of two patients with GERD post OAGB was clearly demonstrated with pH-impedance monitoring data collected and published in a recent study [12]. In this study, 30.23% of patients with acid reflux and 11.64% with mixed reflux (acid and bile) were documented, whereas only 27.9% of patients presented with pure bile (non-acid) reflux. Among patients with pure acid reflux, median DeMeester score and distal esophageal acid exposure time (pH < 4) were 48.95 and 9.65%, respectively, confirming that OAGB patients may be exposed to acidity [12]. The main hypothesis for the development of acid reflux is related to the excess fundal tissue of the gastric pouch responsible for acid secretion by parietal cells. This is probably caused by the formation of an inadequate “large” gastric pouch. Acid secretions in combination with insufficient LES could promote food stasis and indigestion, thus favoring GERD.

Due to the hypothesis of the bile origin of reflux until recently, bile flow diversion has been the procedure of choice in these patients. Indeed, this procedure does not require shortening or reduction in the gastric pouch. The biliopancreatic limb is stapled proximally (3–4 cm) to the gastrojejunal anastomosis [11,12]. Then, an alimentary limb (measuring 60–80 cm) is formed followed by a jejuno-jejunal anastomosis between the biliopancreatic and alimentary limb. However, the recent finding of acid reflux could modify surgical management of impairing and PPI-resistant GERD. Adding a Roux limb to OAGB without shortening the gastric pouch may be effective for bile reflux, but it will not cure acid reflux since the dilated gastric pouch is left untouched [11]. For adequate treatment of acid reflux, another surgical procedure has been proposed requiring complete reconstruction of the OAGB, gastric pouch shortening and realization of the standard Roux-en-Y gastric bypass [11,16]. A decision algorithm (for choosing the type of surgical procedure) based on the results of pre-operative pH-impedance studies has been proposed in a recent study comparing these two aforementioned procedures [11]. This weighs down preoperative assessment by adding costly investigations and makes surgical procedure more challenging in patients with acid reflux. However, given the anticipated results and the increasing complexity of the management of persistent GERD in case of failed surgery, I believe that surgeons should not be reluctant to undertake appropriate assessment in order to achieve the best outcomes for their patients.
